# Smoking Cessation Intervention in a Cardiovascular Hospital Based Clinical Setting

**DOI:** 10.1155/2012/970108

**Published:** 2012-10-14

**Authors:** Zainab Samaan, Barb Nowacki, Karleen Schulze, Patrick Magloire, Sonia S. Anand

**Affiliations:** ^1^Department of Psychiatry and Behavioural Neurosciences, McMaster University, Hamilton, ON, Canada L8S 4L8; ^2^Population Genomics Program, McMaster University, Hamilton, ON, Canada L8S 4K1; ^3^Department of Clinical Epidemiology and Biostatistics, McMaster University, Hamilton, ON, Canada L8S 4K1; ^4^Mood Disorders Program, St. Joseph's Healthcare Hamilton, 100 West 5th Street, Hamilton, ON, Canada L8N 3K7; ^5^Hamilton General Hospital, Hamilton Health Sciences, Hamilton, ON, Canada L8L 2X2; ^6^Population Health Research Institute, Hamilton, ON, Canada L8L 2X2; ^7^Department of Medicine, McMaster University, Hamilton, ON, Canada L8S 4L8

## Abstract

*Introduction*. Smoking is a leading cause of morbidity and mortality globally and it is a significant modifiable risk factor for cardiovascular disease (CVD) and other chronic diseases. Efforts to encourage and support smokers to quit are critical to prevent premature smoking-associated morbidity and mortality. Hospital settings are seldom equipped to help patients to quit smoking thus missing out a valuable opportunity to support patients at risk of smoking complications. We report the impact of a smoking cessation clinic we have established in a tertiary care hospital setting to serve patients with CVD. *Methods*. Patients received behavioural and pharmacological treatments and were followed up for a minimum of 6 months (mean 541 days, SD 197 days). The main study outcome is ≥50% reduction in number of cigarettes smoked at followup. *Results*. One hundred and eighty-six patients completed ≥6 months followup. More than half of the patients (52.7%) achieved ≥50% smoking reduction at follow up. Establishment of a plan to quit smoking and use of nicotine replacement therapy (NRT) were significantly associated with smoking reduction at followup. *Conclusions*. A hospital-based smoking cessation clinic is a beneficial intervention to bring about smoking reduction in approximately half of the patients.

## 1. Introduction

Smoking is a leading cause of premature and preventable death worldwide claiming the lives of over 5 million people each year [1]. Twenty percent of Canadians over the age of 15 years continue to smoke [2]. Lifelong smokers die 10 years earlier than nonsmokers [3]. However, these lost life years can be regained if smoking cessation occurs early [3, 4]. Smoking therefore represents one of the most important modifiable risk factors which when changed can prevent significant mortality and morbidity. Nicotine (a component of cigarettes) is an addictive substance [5] similar in its addictive properties to other addictive substances including opioids and alcohol where nicotine-dependence is manifested by physical withdrawal symptoms and the continued use of smoking to relieve such symptoms [6]. Therefore, attempts to quit smoking can be challenging needing specialized treatments and interventions. Both population and individual tobacco prevention and treatment strategies are required to minimize tobacco use and to reduce the exposure of nonsmokers to secondhand smoke. Effective strategies include increased taxation on tobacco products [7] and banning smoking in public places. In hospital settings, smokers commonly present with life-threatening conditions including CVD (i.e., acute coronary syndromes, stroke, peripheral arterial disease) and as well as exacerbations of lung diseases [8, 9]. Unfortunately acute care settings are often poorly equipped to assist patients to reduce or quit smoking after they are discharged from hospital. This is especially important given that acute hospitalizations strongly motivate patients to quit smoking [10]. 

Several effective interventions are available to aid patients in smoking cessation [11–13]. Nicotine Replacement therapy (NRT), bupropion [14], varenicline [12] and behavioural therapy [15] are all effective treatment options, however, these are rarely offered in hospital settings. Given the significant risks associated with smoking, and the minimal presence of smoking cessation clinics in acute care hospitals, we report the outcomes of a smoking cessation clinic created to serve outpatients at a tertiary care hospital in Ontario, Canada.

The objectives of this study are to (1) characterize the sociodemographic characteristics and health status of patients offered smoking cessation interventions; (2) determine which strategies were associated with a ≥50% reduction in smoking at followup, and (3) explore barriers associated with failure of smoking reduction in this group. 

## 2. Methods 

### 2.1. Study Design

A retrospective chart review of patients attended our Smoking Cessation Clinic (SCC) between June 2005 and November 2010. Data were collected using standardized assessment forms at baseline and follow up clinic visits.

### 2.2. Setting

The SCC is a hospital-based clinic established in 2005 to serve patients with cardiovascular disease who were being seen by physicians in the Hamilton Health Sciences. The SCC was created to address a lack of specific smoking treatment services for cardiovascular patients in Hamilton. Once the smoking cessation clinical service started, additional patients were referred by hospitalists and family physicians. The SCC clinic staff included a tobacco treatment specialist (TTS) guided by 2 cardiovascular physicians and a psychiatrist.

### 2.3. Study Participants

All patients who attended the SCC between June 2005 and November 2010 were included in this study. We report our experience with 253 patients referred to the SCC. 

This study was approved by the institution's Research Ethics Board.

### 2.4. Study Outcome

The main study outcome is the reduction in the number of cigarettes smoked at followup. Patients were divided into 2 groups: those who achieved a ≥50% reduction of smoking or quit completely, and those who did not, in keeping with other studies that treated smoking intervention outcomes as continuous (reduction in number of cigarettes) rather than dichotomized result (cessation or not) [16]. The reduction in the number of cigarettes smoked was included as a successful outcome recognizing that smoking has a dose response effect on most chronic diseases. In addition smoking reduction is associated with future smoking cessation [17] making it an important predictive factor of smoking cessation eventually. Although smoking cessation is considered the ultimate outcome in smoking intervention studies, our sample size is relatively modest to focus on cessation only. Patients were asked about the number of cigarettes smoked at followup and in some patients carbon monoxide (CO) level was measured. However, not all patients had CO measured and therefore this was not used as a study outcome. Thus, smoking reduction and cessation are based on patients' self-report at followup.

### 2.5. Data Source and Measurements

#### 2.5.1. Clinical Assessment

All patients were assessed at the SCC through face-to-face interviews by the TTS. Sociodemographic, medical, and smoking history (obtained through self-report and reviewing the medical chart) and medications were recorded. The patients' current reasons and degree of motivation to quit smoking were also asked and recorded. The Patient Health Questionnaire 2 (PHQ-2) [18] was administered to each patient as a screen for depression and an affirmative answer to either question of PHQ-2 was considered to be a positive screen of depression. In addition, among patients who reported a prior psychiatric history, we inquired as to whether they were receiving active followup with psychiatric services or from their family practitioners for these disorders as well as current psychotropic medications. 

The level of tobacco dependence was assessed using the Fagerstrom questionnaire [19] and attitudes to smoking and quitting were explored using a set of questions adopted from the Centre of Addiction and Mental Health, Toronto, and University of Ottawa Heart Institute smoking cessation programs. A social disadvantage score was assigned to each patient using the Social Disadvantage Index (SDI) which is based on sociodemographic characteristics (i.e., annual household income, employment status, marital status), the scoring of which ranges from 0 to 5, with 5 reflecting high social disadvantage [20]. 

#### 2.5.2. Smoking Cessation Individualized Plan

 Patients received information on different smoking cessation aids including NRT, bupropion, varenicline, or a combination of NRT plus bupropion or NRT plus varenicline. Many patients could not afford NRT or other pharmacological agents, and for these individuals the TTS provided these agents free of charge using clinic samples. Patients were encouraged to set a “quit date.” They were also offered the option of attending the SCC on a regular basis to receive counselling and support based on motivational interviewing by the TTS. This model was broadly used with emphasis on negotiation and flexibility to accommodate personal circumstances [21]. All counselling provided by the same therapist at baseline and follow up visits. 

#### 2.5.3. Followup

The schedule of followup was flexible, ranged from 1 to 4 visits per month and largely dependent on individuals needs and circumstances using an “open door policy” that was adopted by the clinic to maximize adherence. At 6 months followup, an assessment of progress was made to identify which individuals stopped smoking and reduced their tobacco intake. The followup assessments occurred at a minimum of 6 months and up to 18 months. 

#### 2.5.4. Behavioural Management Strategies

 Behavioural modification management was based on the model of motivational interviewing. Individuals received frequent (1–4 sessions per month) counselling including individual or couples counselling when both partners were interested in quitting smoking. The focus of the counselling was on behavioural change, addressing ambivalence and helping patients reach their goal of reducing and quitting smoking. Individuals were encouraged to “follow the process and not the outcome” and were also encouraged to consider a “practice makes perfect” model. Individuals were therefore encouraged to adhere to the process and move from planning to trying and eventually quitting in keeping with motivational interviewing guiding principles [22]. Problem solving strategies and discussions regarding individual challenges, secondhand smoking, and temptation due to family or others around them were also used during counselling.

### 2.6. Study Size

All patients attended the clinic were potential study participants. No sample size or power estimations were performed. 

## 3. Statistical Methods

Statistical analyses were performed using SAS, version 9.1 (SAS Institute, Cary, NC, USA). Means, standard deviations, and proportions are presented for descriptive variables. Between-group comparisons were made using ANOVA for continuous variables and chi-square tests for categorical variables. Univariate and multivariate logistic regression models adjusted for age and sex were used to determine the independent predictors of smoking reduction at followup. In the univariate models, we used smoking reduction/cessation as the outcome variable and tested each of the following variables separately: age; sex; marital status; setting a quit date; NRT; lack of will/confidence; concerns regarding social reasons, stress, weight gain, and finances; Fagerstrom dependence score; positive depression screen based on PHQ-2. Each factor that achieved a *P* value of 0.10 or less in the univariate models, age and sex were included in the multivariate logistic regression analysis. Backwards selection methods simplified the model to identify independent predictors of successful smoking reduction/cessation. The study outcome was defined as complete smoking cessation or ≥50% reduction in the number of cigarettes smoked at the final followup. 

## 4. Results

### 4.1. Participants

**Figure 1:**
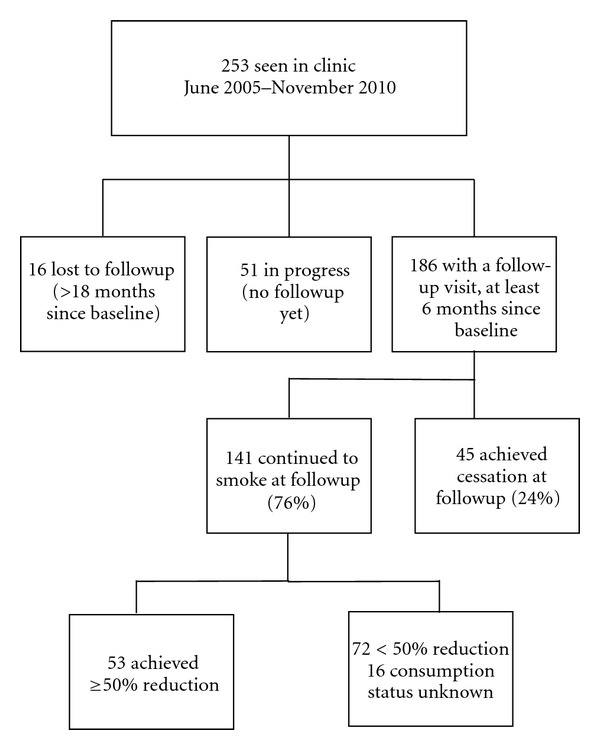
Study participants flow diagram.

Two hundred and fifty-three outpatients were seen at the SCC between June 2005 and November 2010. One hundred and eighty-six patients completed a follow up visit (minimum 6 months), 51 patients are still in progress (no follow up assessment has been completed yet), and 16 individuals dropped out from the clinic followup. (i.e., no follow up within 18 months of baseline).  Figure 1 presents the study participants flow diagram.

### 4.2. Sociodemographics Characteristics

Individuals' characteristics are presented in Table 1. The mean age was 54.1 years (SD 12.0) and 54.5% were men. More than 50% of the patients scored 3 or more on the SDI reflecting moderate-to-high social disadvantage. 

### 4.3. Health Status of Study Participants

The majority of patients (76.7%) reported history of CVD as seen in Table 2. A third of patients reported a psychiatric history and 48% screened positive for depression on PHQ-2 questionnaire. 

### 4.4. Primary Outcome: Smoking Status at Followup

**Figure 2:**
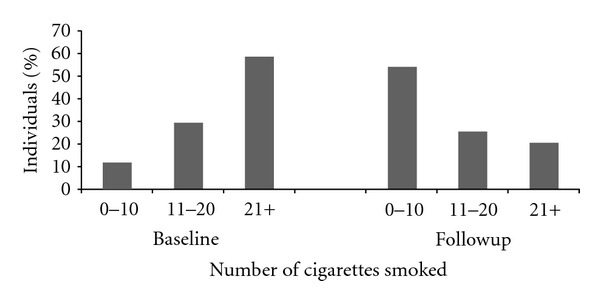
Comparison of number of cigarettes smoked at baseline and followup.

More than half of patients (52.7%; 95% CI: 45.5, 59.9) were successful in achieving ≥50% reduction of number of cigarette smoked at followup. The majority of patients used NRT and most of these patients received NRT from the clinic.  Table 3 presents the proportion of each pharmacotherapy used in the treatment of the clinic patients. Univariate analyses (Table 4) showed that individuals who had a quit date (OR 4.22, 95% CI: 1.78, 9.99, *P* = 0.001,) and used any form of NRT (OR 2.43, 95% CI: 1.31, 4.52, *P* = 0.005) were more likely to succeed in achieving ≥50% reduction of number of cigarettes at followup.  Figure 2 also shows a significant reduction in the number of cigarettes smoked at followup compared to baseline (*P* < 0.0001), while other factors including lack of will/confidence (OR 0.39, 95% CI: 0.22, 0.71, *P* = 0.002) and a high Fagerstorm score of nicotine dependence (OR 0.43, 95% CI: 0.23, 0.79, *P* = 0.007) were associated with failure to reduce smoking. 

Factors such as number of previous quit attempts (*P* = 0.20), level of income (*P* = 0.87), number of contacts with clinic (*P* = 0.41), and duration of followup (*P* = 0.23) were not associated with smoking reduction.

**Figure 3:**
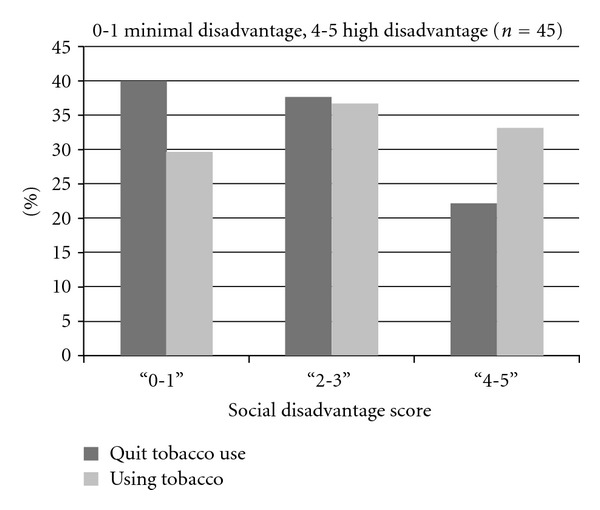
Smoking cessation by social disadvantage score.

A subgroup analysis of smoking cessation (*n* = 45) showed that patients with high SDI score (4 or 5) were less likely to quit smoking compared to patients in the lowest score category. While not statistically significant, a trend (trend test *P* = 0.10) towards successful reduction in the absence of social disadvantage was observed (Figure 3).

Multivariate analysis (Table 5) showed that individuals using NRT were twice as likely to succeed at smoking reduction/cessation at followup (OR 2.15, 95% CI: 1.11, 4.19, *P* = 0.024). On the other hand, individuals who expressed concerns about quitting due to lack of will/confidence (OR 0.46, 95% CI: 0.24, 0.87, *P* = 0.016), social barriers (i.e., exposure to secondhand smoking by family and friends, temptation when others smoke around them, and socializing with peers who smoke) (OR 0.51, 95% CI: 0.27, 0.99, *P* = 0.045), and fear of weight gain (OR 0.50, 95% CI: 0.26, 0.97, *P* = 0.039) were less likely to succeed. Sensitivity analysis using smoking cessation only as an outcome showed similar results (data not shown).

## 5. Discussion 

We describe the usefulness of smoking cessation treatment in clinical setting serving patients with cardiovascular disease. Our experience showed that such services are helpful and urgently needed as a secondary prevention for cardiovascular disease. In addition, our study showed that patients attended the clinic had several comorbidities including psychiatric and social disadvantage making them additionally vulnerable group of patients. Overall, more than half of the patients (52.7%; 95% CI: 45.5, 59.9) were able to reduce smoking at followup with the aid of NRT and behavioural intervention administered by a TTS. Factors that were associated with failure to reduce smoking in this group included high nicotine dependency score and lack of will and confidence to quit smoking.

Despite the social disadvantage including low income of many of the patients referred to this clinic, these factors were not statistically significantly associated with number of cigarette smoked at followup, although a trend was seen towards a successful reduction in the absence of social disadvantage. It is likely that the reason for lack of significance here is that the majority of these patients received NRT from the clinic thus eliminating the concerns regarding cost of NRT. The clinic aim was to support patients in all aspects of smoking reduction including lifting the financial burden based on our knowledge of cost and cost comparisons between smoking and NRT (we estimated the cost of smoking locally produced “aboriginal reserve cigarettes” commonly used in our region, per week to be $21 compared to the cost of NRT at $91/week). 

Several previous studies have shown that interventions to promote smoking cessation can be effective including group and individual behavioural therapy, physician advice, NRT, bupropion, and nursing interventions [23]. Despite the availability of such interventions, smoking cessation remains a significant challenge to many individuals at risk. Previous studies have shown a significant impact of inpatient smoking cessation programs on patients with CVD. Smoking cessation (self-reported) was achieved in 46% of patients at 6 months after myocardial infarction (MI), with greater odds of cessation for those offered inpatient smoking cessation services [24]. 

Even though effective treatments for smoking cessation are available, several barriers exist in the process of smoking cessation for many individuals, and these can be intrinsic, including the psychological factors such as lack of will and confidence, or extrinsic, such as financial means and the lack of dedicated service within hospital settings where they are needed most to help vulnerable patients. 

## 6. Limitations

Our study has some limitations including the observational nature of the study design and the inclusion of high risk motivated patients seen in a hospital setting. Thus while our findings are internally valid, they may not be generalizable to the nonhospital-based population of smokers. In addition, the exact duration of smoking cessation/reduction among our patients is unknown and dependent on self-report. No objective measure of smoking reduction such as CO level was used in all patients, and this may have led to an overestimation of the degree of smoking reduction due to the social desirability bias often inherent in the self-reporting of health behaviours to health care practitioners. There were also very few patients who used other forms of pharmacotherapy such as varenicline and bupropion and therefore the effect of these medications on smoking cessation cannot be assessed in this study. However the use of varenicline in patients with CVD may be limited due to a potential risk of increased CVD events [25]. Barriers to smoking cessation we described might also be confounded by the relatively high proportion of patients with reported depressive symptoms, which may influence patients' perception of social difficulties. Nevertheless at a minimum our experience provides more evidence that acute care centres with large cardiac and psychiatric populations should create and sustain a formal smoking cessation program to assist their patients in improving their long-term health through smoking cessation. 

Finally, this is an observational study of clinical service provision in a real world setting. The impact of our intervention is likely influenced by the self-selection of motivated patients and the personal attention received from our TTS. Thus while our associations are internally valid, the generalizability of our findings to other settings merits discussion.

## 7. Conclusion

A hospital-based SCC is an effective intervention to bring about smoking reduction in approximately half of the patients. Policy level interventions to aid smoking cessation should include supporting hospital-based SCC, where they are needed most, and provision of NRT at low cost. These factors will likely aid in improving the immediate goals of enhancing smoking cessation and will also have important long-term health and economic benefits for the patients and their community.

## Figures and Tables

**Table 1:** Sociodemographic details of patients attended smoking cessation clinic in Hamilton, Ontario, Canada.

Variable	Description	Overall *N* = 253*	With followup *N* = 186	Dropped out *N* = 16
Age (years)	19–44	46 (18.2%)	30 (16.1%)	2 (12.5%)
	45–54	80 (31.6%)	59 (31.7%)	6 (37.5%)
	55–64	75 (29.6%)	59 (31.7%)	6 (37.5%)
	65+	51 (20.2%)	37 (19.9%)	2 (12.5%)

Sex	Male	138 (54.5%)	104 (55.9%)	11 (68.8%)
	Female	115 (45.5%)	82 (44.1%)	5 (31.3%)

Marital status	Never married	24 (9.5%)	16 (8.6%)	1 (6.3%)
	Currently married	127 (50.2%)	102 (54.8%)	7 (43.8%)
	Common law/living partner	22 (8.7%)	8 (4.3%)	2 (12.5%)
	Separated	16 (6.3%)	11 (5.9%)	2 (12.5%)
	Divorced	38 (15.0%)	29 (15.6%)	1 (6.3%)
	Widowed	19 (7.5%)	14 (7.5%)	2 (12.5%)

Education	None	1 (0.4%)	1 (0.5%)	0 (0.0%)
	1–8 years	27 (10.7%)	22 (11.8%)	2 (12.5%)
	9–12 years	109 (43.1%)	79 (42.5%)	7 (43.8%)
	Trade school	31 (12.3%)	24 (12.9%)	3 (18.8%)
	College/university	72 (28.5%)	48 (25.8%)	3 (18.8%)

Income in $	0–29.9K	92 (36.4%)	66 (35.5%)	6 (37.5%)
	30–39.9K	26 (10.3%)	18 (9.7%)	2 (12.5%)
	40–49.9K	25 (9.9%)	18 (9.7%)	1 (6.3%)
	50–69.9K	31 (12.3%)	22 (11.8%)	2 (12.5%)
	>70 K	50 (19.8%)	39 (21.0%)	3 (18.8%)

Employment	Currently employed	116 (45.8%)	83 (44.6%)	5 (31.3%)

SDI^†^	0 = none	48 (19.0%)	38 (20.4%)	3 (18.8%)
	1	33 (13.0%)	22 (11.8%)	1 (6.3%)
	2	39 (15.4%)	29 (15.6%)	1 (6.3%)
	3	55 (21.7%)	40 (21.5%)	6 (37.5%)
	4	39 (15.4%)	27 (14.5%)	3 (18.8%)
	5 = high	39 (15.4%)	30 (16.1%)	2 (12.5%)

*51 in progress. ^†^SDI Social Disadvantage Index.

**Table 2:** Medical history of patients attended smoking cessation clinic.

Medical history categories	Description	Overall *n* = 253	Followup *n* = 186
CVD*			
PAD	Peripheral arterial disease	95 (38.3%)	73 (40.1%)
CAD	Coronary artery disease	121 (48.2%)	92 (49.7%)
CBVD	Cerebrovascular disease	38 (15.08%)	32 (17.3%)
Other	Other CVD	137 (54.15%)	102 (54.84%)
Surgery	Surgery	20 (7.9%)	16 (8.6%)
Endometabolic	Abnormal lipids	171 (67.6%)	129 (69.4%)
	Diabetes	48 (19.0%)	37 (19.9%)
Neurological	Seizures	12 (4.7%)	10 (5.4%)
	Head injury	35 (13.8%)	26 (14.0%)
Psychiatric	Eating disorders	5 (2.0%)	3 (1.6%)
	History of psychiatric illness	79 (31.2%)	63 (33.9%)
Cancer	Cancer	21 (8.3%)	16 (8.6%)
Dermatologic	Skin rash	42 (16.6%)	32 (17.2%)
Respiratory	COPD^*∧*^/asthma	65 (25.7%)	46 (24.7%)
Hepatic	Hepatic insufficiency	15 (5.9%)	10 (5.4%)
Renal	Renal insufficiency	21 (8.3%)	14 (7.5%)
Other	Other	149 (58.9%)	103 (55.4%)

*CVD: cardiovascular disease.

^*∧*^COPD: chronic obstructive pulmonary disease.

Numbers do not add to 100% due to overlap and comorbidity of several illnesses in same individuals.

**Table 3:** NRT and other smoking cessation treatment uptake by patients attended smoking cessation clinic.

Treatment options used	Use of tobacco products
	*n* (%)
Individuals with followup	186
Individuals who prepared a quit strategy	154 (82.8%)
Bupropion	39 (21.0%)
Any NRT	121 (65.1%)
(i) Patch	80 (43.0%)
(ii) Inhaler	78 (41.9%)
(iii) Gum	47 (25.3%)
(iv) Lozenges	9 (4.8%)
Varenicline	12 (6.5%)
Bupropion + NRT	34 (18.3%)

**Table 4:** Univariate analysis of smoking reduction/cessation.

Variable	OR	95% CI	*P*
Age	1.02	0.99, 1.05	0.117
Male	1.73	0.96, 3.10	0.067
Married/common law	1.53	0.84, 2.80	0.164
Individual set a quit date	4.22	1.78, 9.99	0.001
NRT	2.43	1.31, 4.52	0.005
Lack of will/confidence	0.39	0.22, 0.71	0.002
Concerns social reasons	0.37	0.20, 0.67	0.001
Concerns stress	0.52	0.29, 0.94	0.031
Concerns weight gain	0.44	0.25, 0.80	0.007
Concerns financial	0.55	0.29, 1.02	0.056
High Fagerstrom dependence score	0.43	0.23, 0.79	0.007
Positive depression screen	0.48	0.26, 0.89	0.020

**Table 5:** Multivariate analysis of smoking cessation/reduction.

Variable	OR	95% CI	*P*
Age	1.01	0.98, 1.04	0.358
Male	1.29	0.66, 2.50	0.460
NRT	2.15	1.11, 4.19	0.024
Lack of will/confidence	0.46	0.24, 0.87	0.016
Concerns social reasons	0.51	0.27, 0.99	0.045
Concerns weight gain	0.50	0.26, 0.97	0.039
